# Insights into Microbiota–Vaccine Crosstalk in Humans: Mechanisms, Modulators, and Translational Horizons

**DOI:** 10.3390/vaccines14090791

**Published:** 2026-09-09

**Authors:** Ahmad R. Shakri, Sidharth P. Mishra, Sarina Lawless, Saswati Pani, Priyanka Mishra, Courtney L. Page, Gaurav Dutta, Chanchal Sharma

**Affiliations:** 1 Department of Internal Medicine, University of South Florida Morsani College of Medicine, Tampa, FL 33612, USA; 2 USF Center for Microbiome Research, Microbiomes Institute, University of South Florida, Tampa, FL 33612, USA; 3 Center for Excellence of Aging and Brain Repair, Department of Neurosurgery, Brain and Spine, University of South Florida, Tampa, FL 33612, USA; 4 USF Health, University of South Florida Morsani College of Medicine, Tampa, FL 33602, USA; 5 Immunology Laboratory, Fish Health Management Division, CIFA, Indian Council of Agricultural Research, Bhubaneswar 751002, Odisha, India; 6 Department of Biotechnology, GITAM School of Sciences, GITAM University, Visakhapatnam 530045, Andhra Pradesh, India; 7 Department of Microbiology and Immunology, Wake Forest University School of Medicine, Winston-Salem, NC 27157, USA; 8 Department of Animal Science, University of Connecticut, Storrs, CT 06269, USA; 9 School of Chemistry, Chemical Engineering and Biotechnology, Nanyang Technological University, Singapore 639798, Singapore

**Keywords:** vaccine, microbiota, short-chain fatty acids, immune modulation, dysbiosis, probiotics, gut–vaccine crosstalk, immunogenicity

## Abstract

Vaccine responses differ substantially among individuals and across populations. Although factors such as age, genetics, and vaccine type are recognized contributors, they do not fully explain this heterogeneity. Emerging evidence suggests that the human microbiota, particularly the gut microbiota, may modulate immune responses to vaccination and represents a potentially modifiable component of immunity. This review integrates data from human studies, microbiota-targeted clinical trials, and experiments using germ-free and humanized models to clarify the mechanisms underlying microbiota–immune system interactions during vaccination. Identified mechanisms include pattern-recognition receptor signaling, modulation of innate immune activation, regulation of germinal-center responses, maintenance of mucosal barrier integrity, and the influence of microbial metabolites on T and B lymphocytes. The relevance of these pathways varies by age, developmental stage, and vaccine platform, including live-attenuated, inactivated, subunit, viral-vector, and mRNA vaccines. Additional factors such as diet, antibiotic exposure, infections, medications, and environmental or social determinants also affect both the microbiota and vaccine outcomes. Current research explores approaches to improve vaccine potency through microbiota modulation using probiotics, prebiotics, synbiotics, postbiotics, and engineered microbes, though clinical results remain inconsistent. A greater understanding of microbiota–vaccine interactions may enable personalized immunization strategies; however, further research is required to establish causality and identify actionable microbial targets. Longitudinal studies using multi-omics, advanced cellular analyses, robust clinical trials, and in silico modeling are essential to determine whether microbiome-based interventions can improve vaccine efficacy and durability.

## 1. Introduction

Vaccination remains one of the most effective public health interventions, saving millions of lives every year. Nonetheless, immune responses to vaccines differ markedly depending on geographic location, age, and other factors. Though genetics, age, comorbidities, and vaccine type are established determinants, recent research points to the human microbiota as an additional influential factor in vaccine efficacy [1,2]. The microbiota, a lively ecosystem within the body, shapes immune function before, during, and after vaccination [1,3,4]. Among all microbial communities, the gut microbiota has been most extensively investigated in relation to vaccine responses. Human studies have demonstrated associations between gut microbial composition and individual responses to vaccines such as rotavirus, polio, influenza, and SARS-CoV-2 [5,6]. Both human and animal studies indicate that the antibiotic-induced disruption of the gut microbiota correlates with altered vaccine responses. Collectively, these results indicate that microbiota composition and activity influence immune responses to vaccination, although the basic mechanisms and their value in real-life situations remain incompletely understood.

Mechanistic studies offer biological frameworks by which the microbiota might affect vaccine-induced immunity. Microbial molecular patterns interact with pattern-recognition receptors, such as Toll-like receptors (TLRs) and nucleotide-binding oligomerization domain (NOD)-like receptors, and affect the innate immune response, antigen presentation, and subsequent priming of the adaptive immune system [7,8]. At the same time, metabolites derived from the microbiota, such as short-chain fatty acids (SCFAs), tryptophan metabolites, and secondary bile acids, are able to regulate the integrity of the epithelial barrier, dendritic cell function, and the differentiation of T cells and B cells by means of metabolic and epigenetic pathways [1,9,10]. However, because much of such mechanistic evidence comes from germ-free, antibiotic-treated, gnotobiotic, or other experimental settings, it must be handled with caution when applied to human vaccination [8]. As in the gut, microbial communities in the skin and respiratory tract may also affect local immune environments relevant to intradermal and mucosal vaccination [11,12].

The vaccine platform could also affect the importance of microbiota–immune interactions. Since live-attenuated vaccines, especially those given orally or intranasally, rely heavily on mucosal surface integrity and local immune activation, they may be especially vulnerable to dysbiosis or environmental enteric dysfunction [6]. Although inactivated and subunit vaccines do not depend as directly on mucosal colonization, they can still be influenced by the initial immune environment formed by microbial products and metabolites [8,9,11]. Decreased microbiota diversity has also been associated with reduced neutralizing antibody responses after vaccination against coronavirus disease 2019 (COVID-19) with mRNA vaccines [13,14]. However, the extent to which the microbiota affects responses to different vaccine platforms or adjuvants remains unclear.

Environmental and clinical factors can further change the gut microbial ecosystem. Dietary patterns and micronutrient levels affect microbial composition, metabolite production, intestinal inflammation, and barrier stability [15,16,17], though direct evidence linking diet-driven microbiome configurations to human vaccine immunogenicity remains limited. Geographic and social and economic factors also affect microbial ecology and vaccine responses through their interrelated effects on nutrition, sanitation, infection burden, access to medical care, and exposure to antimicrobials [10,11]. Interventions that target the microbiota are therefore being considered as possible ways to modify vaccine responsiveness. Some studies using probiotics and synbiotics indicate effects on vaccine-specific immune responses, but findings remain varied and depend on the strain, vaccine, population, and study design [6,18,19]. Other methods, such as postbiotics, fecal microbiota transplantation (FMT), and engineered commensals, remain largely experimental and require more confirmation for use with vaccines.

In this review, evidence is evaluated based on study design and clinical relevance. Human randomized or interventional studies are differentiated from observational associations, while germ-free, gnotobiotic, and antibiotic-treated animal models, as well as in vitro studies, are primarily referenced for mechanistic insight. General microbiome–immune interactions are considered biologically plausible pathways when direct vaccine-specific evidence is lacking; however, associations between microbial characteristics and vaccine outcomes are not regarded as causal unless substantiated by interventional data.

Although interest in microbiota-informed vaccinology is growing, important knowledge gaps remain. Human studies remain mainly observational, limiting the ability to draw causal conclusions, and longitudinal multi-omics analyses of vaccination remain relatively rare. Mechanistic evidence is especially limited for newer vaccine platforms, such as mRNA and viral-vector vaccines. Preterm infants, newborns, older people, immunocompromised individuals, and patients with chronic inflammatory or metabolic disorders are also still underrepresented in microbiota–vaccine studies [4,11]. Translational tools that can predict microbiome-associated vaccine responses are still in early development, and regulatory systems for microbiome-based adjuvants or therapeutics remain immature. Overcoming these limitations through longitudinal human studies, well-designed interventions, and mechanistic validation might help determine whether the microbiota acts as a causal contributor, is a modifiable therapeutic target, or is merely a biomarker of wider host and ecological influences. Such progress could allow more precise strategies to improve the magnitude, durability, and consistency of vaccine-induced protection across populations.

The review was based on a systematic search of PubMed/MEDLINE, Scopus, Web of Science, and Google Scholar for studies published between 2010 and 2026 that examined interactions between the gut microbiota and vaccines. The search used combinations of the following terms: “gut microbiota”, “gut microbiome”, “vaccination”, “vaccine immunogenicity”, “antibody response”, “seroconversion”, “probiotics”, “prebiotics”, “synbiotics”, “postbiotics”, “short-chain fatty acids”, “microbial metabolites”, “antibiotics”, “germ-free”, “gnotobiotic”, “trained immunity”, and terms pertaining to various vaccines such as influenza, rotavirus, polio, BCG, COVID-19, and mRNA vaccines. For vaccine immunogenicity and clinical outcomes, we prioritized human observational and interventional studies, while including animal, germ-free, gnotobiotic, antibiotic-use, and in vitro studies mainly to provide mechanistic support. Any studies that were not related to the microbiota, immune regulation, or vaccination; duplicate reports; non-scientific publications; and studies that did not contain enough methodological or outcome information were excluded. We preferred original peer-reviewed studies, using reviews mainly to offer context and help identify relevant primary literature. The study considered preprints selectively for emerging topics when peer-reviewed evidence was lacking and noted them as such. Because this was a narrative review, study selection was guided by scientific relevance and contribution to the conceptual framework rather than by formal systematic-review or meta-analytic criteria.

## 2. Mechanistic Basis of Gut–Vaccine Crosstalk

The gut microbiota influences how vaccines work through a coordinated system of signals that primes innate immune sensors, shapes the fate of adaptive lymphocytes, strengthens mucosal structure, and establishes epigenetic programs in myeloid cells. When the pattern recognition receptors (such as the TLRs and NLRs) detect ligands from the normal flora, dendritic cells and macrophages acquire a greater ability to present antigen and produce cytokines in a regulated manner, thus establishing the ‘immune set point’ into which vaccines are introduced [9,20,21]. At the same time, the metabolic pathways of the SCFAs, the indoles derived from tryptophan, and the secondary bile acids exert their effects by acting on G protein-coupled receptors (GPCRs) and nuclear receptor pathways to promote class-switch recombination, improve the quality of germinal centers, and enhance the durability of memory responses. Some postbiotics can also act as safe, microbe-derived adjuvants [22,23]. The state of the barrier and antigen transport via microfold (M) cells, Peyer’s patches, and the more extensive gut-associated lymphoid tissue (GALT) also determine the balance between effective priming and unwanted bystander inflammation. This is particularly clear in mucosal (for example, oral) vaccination [4,6,24]. However, it is important to distinguish mechanisms shown directly in vaccination studies from broader microbiome–immune pathways that offer biological plausibility. Several of the mechanisms mentioned below, particularly those relating to microbial metabolites, epigenetic regulation, and lymphocyte differentiation, are mainly supported by experimental or non-vaccination studies and have therefore not yet been directly validated in vaccinated human populations.

The innate immune system’s priming and antigen presentation: This process is the first stage at which the microbiota can affect vaccination. Commensal microorganisms continuously supply small amounts of microbial-associated molecular patterns, such as flagellin, peptidoglycan, lipoteichoic acid, and other cell-wall components, which pattern-recognition receptors (PRRs), especially Toll-like receptors (TLRs) and nucleotide-binding oligomerization domain-like receptors (NLRs), detect [25,26]. This constant microbial signaling helps keep dendritic cells (DCs), macrophages, and other innate immune cells responsive yet under control, thus setting an ‘immune set point’ before vaccination. Experimental studies show that microbiota-dependent signaling via PRRs can alter the expression of MHC class II and co-stimulatory molecules and regulate cytokine production by antigen-presenting cells [27,28,29], offering a plausible mechanism by which ongoing microbial signaling might influence vaccine priming.

Microbial conditioning extends beyond direct PRR interactions. Microbial metabolites affect the metabolic and epigenetic state of antigen-presenting cells and, in turn, can alter their response to vaccine antigens. SCFAs, tryptophan-derived metabolites, and secondary bile acids transmit signals via GPCRs, the aryl hydrocarbon receptor (AhR), the farnesoid X receptor (FXR), TGR5, and related pathways, influencing inflammation levels and APC function [30,31,32]. However, these effects are context-dependent; microbial signaling must promote effective immune activation without causing excessive basal inflammation that might interfere with antigen processing or adaptive priming.

The intestinal barrier adds another layer of control, especially for mucosal vaccines. Mucus, the tight junctions, secretory IgA, M cells, Peyer’s patches, and other parts of the gut-associated lymphoid tissue all play a role in controlling the interaction between antigens in the lumen and immune cells. If dysbiosis, malnutrition, or an enteric infection occurs, this interface can be disrupted, leading to chronic inflammation that may impair antigen presentation and reduce responses to mucosal vaccines [4,33]. Hence, the microbiota affects the first stage of vaccination by influencing microbial sensing, antigen-presenting cell function, inflammation levels, and mucosal antigen transport.

Microbiota Regulation of Vaccine-Induced Adaptive Immunity: Effective vaccination requires the transition from innate antigen recognition to antigen-specific T- and B-cell responses. The gut microbiota contributes to this transition by influencing DC-derived cytokines and the metabolic signals that determine lymphocyte activation and differentiation. Microbial products can shape the balance among Th1, Th2, Th17, T follicular helper (Tfh), regulatory T-cell (Treg), and CD8^+^ T-cell responses, thus affecting the cellular phenotype generated following immunization [34,35].

For instance, microbial ligands and metabolites derived from tryptophan can affect T-cell differentiation via signaling that depends on PRRs and AhR. In contrast, secondary bile acids and SCFAs can promote regulatory programs that suppress excessive inflammation [30,31,32,36,37,38]. This regulation is important because, for a vaccine to be effective, an appropriate balance between effector cell activation and immune regulation is needed, not maximal inflammatory stimulation. Microbial conditioning may also affect CD8^+^ effector and recall responses, as well as the breadth of the T-cell receptor repertoire [8,34,35]. As a result, variations in microbial composition and metabolic activity before vaccination may explain differences between individuals in the strength and phenotype of the cellular immunity induced by vaccination.

Antibody Production and Germinal-Center Responses: The microbiota may also affect vaccine efficacy by influencing germinal-center reactions, B-cell differentiation, immunoglobulin class switching, and plasma cell generation. Signals from microorganisms that reach secondary and mucosal lymphoid tissues can affect interactions among B cells, Tfh cells, and antigen-presenting cells that determine the affinity, isotype, and duration of the antibody response. In various experimental models involving germ-free animals or those treated with antibiotics, impaired germinal-center formation and diminished vaccine-specific IgG or IgA responses are commonly observed; however, restoring microbial signals can recover these aspects of humoral immunity [39,40].

Microbial metabolites play an important role in linking intestinal ecology to these humoral responses. Acetate, propionate, and butyrate can affect immune cell metabolism and chromatin accessibility and have been linked to B-cell differentiation, class-switch recombination, and plasma cell function [41,42,43,44]. These findings provide a plausible mechanism for the association between metabolically active commensal communities and more robust vaccine-induced antibody responses. Yet the extent and direction of these effects will likely depend on metabolite concentration, anatomical compartment, vaccine platform, and the host’s immune status; thus, results from experimental systems cannot be assumed to translate directly to humans.

Microbiota, Trained Immunity, and Durability of Vaccine-Induced Memory: After the initial immune response, the microbiota may affect how long vaccine protection lasts by promoting both adaptive memory and more prolonged functional states in innate immune cells. Microbial ligands and metabolites can trigger metabolic and epigenetic reprogramming in monocytes and macrophages, leading to trained-immunity phenotypes characterized by a modified response to later stimulation [45,46]. Changes in histone modifications, such as H3K4me3 and H3K27ac, together with the restructuring of glycolytic and oxidative metabolic pathways, can explain how previous microbial exposure influences responses during vaccination.

The microbiota may also help maintain antigen-specific immunity by promoting the differentiation of memory T-cells and ensuring the metabolic fitness of long-lived plasma cells in bone-marrow niches [44,47,48]. Supporting this idea, antibiotic-induced disruption of the microbiota has been shown to alter early transcriptional and plasmablast responses following vaccination. Conversely, experimental recolonization or restoration of microbial metabolites can reverse some of these immune defects [39,49]. Taken together, these findings show that the microbiota may affect both the size of the initial vaccine response and its ongoing phase.

SCFAs are an excellent example of how a single group of microbial metabolites can affect various aspects of vaccine immunity. Rather than operating via a single pathway, they can signal through GPCRs and inhibit histone deacetylases, influencing APC activation, T-cell differentiation, B-cell metabolism and class switching, and the development or maintenance of immune memory. It is therefore appropriate to consider their effects as part of an integrated immunometabolic network rather than as separate mechanisms acting at each stage of vaccination. Indeed, much of this mechanistic framework has been established using germ-free, antibiotic-treated, gnotobiotic, or other experimental models; it remains important to determine how much these pathways contribute to vaccine responsiveness in humans.

## 3. Human Development Stages and Vaccination: Influence of Gut Microbiota

The response to vaccination varies greatly across life stages because the immune system and gut microbiota develop and restructure concurrently. Differences in age-associated microorganisms can affect vaccination not only by altering the microbiota’s taxonomic makeup but also by changing its metabolic activity, epithelial barrier function, signaling via pattern-recognition receptors, and baseline inflammation. These factors could, in turn, affect antigen presentation, T- and B-cell activation, germinal center reactions, antibody production, and immune memory formation. Therefore, the microbiota may contribute to the different immunological conditions that exist in early life, during adolescence, in adulthood, and in old age, conditions that either promote or hinder vaccine-induced immunity. This relationship is especially significant at the two extremes of age, since the microbial immaturity seen in infancy and the dysbiosis linked to aging occur alongside reduced or less durable responses to many vaccines.

Prenatal and Maternal Influences: The mother’s microbial environment during pregnancy and after birth may influence the immunological setting in which the newborn’s response to vaccination later develops. Although it is still debated whether the fetus itself becomes colonized, products and metabolites from the mother’s microbes can reach the fetal compartment and affect immune systems that are still developing [50,51]. Short-chain fatty acids and metabolites derived from tryptophan can regulate innate immune activity and influence T-cell differentiation, suggesting a possible link between maternal microbial metabolism and early immune competence. Maternal dysbiosis, which can be caused by an unbalanced diet, antibiotic exposure, cancer, obesity, or metabolic disturbances during pregnancy, may therefore modify the microbial and metabolic signals available during fetal and neonatal immune development [52,53,54,55]. After birth, the mother’s microbiota continues to affect the infant’s immune system through vertical transmission of microbes and breastfeeding. These processes could affect the establishment of microbial communities that support development of the mucosal barrier, function of antigen-presenting cells, and maturation of B and T cells when vaccines such as BCG, hepatitis B, rotavirus, and others are given. During this period, protection is also provided by immunoglobulins transferred from the mother through the placenta and breast milk, although these may also interact with the infant’s developing immune response [56,57]. Hence, interactions between the mother’s microbiota and her immune system may influence vaccination indirectly by shaping the microbial and immunological conditions in which the newborn’s vaccine responses develop, rather than serving as an independent factor determining vaccine efficacy.

Infancy and Early Childhood: Infancy and early childhood are a particularly significant time for the interaction between the microbiota and vaccines because the microbial community develops at the same time as the rapid maturation of both the innate and adaptive immune systems. Delivery method, breastfeeding, diet, antibiotic use, and environmental exposure all affect early microbial development [58]. For instance, breastfeeding leads to a microbial composition dominated by *Bifidobacterium* through human milk oligosaccharides. In contrast, disruption of normal microbial maturation can result in the proliferation of facultative or potentially pro-inflammatory organisms. Functionally, these differences may affect vaccine responses by altering microbial metabolite production, intestinal barrier maturation, innate conditioning mediated by pattern recognition receptors, and subsequent antigen presentation.

There is a greater presence of *Bifidobacterium longum subsp. infantis* and other related organisms, which have been linked to more effective reactions to the rotavirus, BCG, and oral polio vaccines [59]. In contrast, delayed microbiota maturation and increased Enterobacteriaceae levels have been linked to poorer vaccine responses [60]. A reasonable mechanism is that a metabolically balanced microbiota in early life sends microbial signals that promote dendritic cell maturation and appropriate T-cell assistance, helping activate B cells, induce class switching, and drive antibody production. By contrast, dysbiosis together with ongoing intestinal inflammation might result in an immune environment in which antigen-specific responses are generated less efficiently.

This mechanism is especially pertinent to environmental enteric dysfunction, since defective epithelial integrity, microbial translocation, and chronic intestinal inflammation can disrupt antigen uptake and the organization of the mucosal immune system [61]. These changes might impair the body’s response to oral vaccines, which rely on effective sampling of antigens through the intestinal mucosa and triggering local IgA responses. Early-life responsiveness to vaccines may therefore depend not only on whether particular microbial groups are present but also on the functional maturation of the microbiota–barrier–immune axis. As a result, breastfeeding, avoiding unnecessary antibiotic use, and using appropriately chosen probiotics or synbiotics are currently being studied as ways to support this developmental period [62,63].

Adolescence: During adolescence, the microbiota tends to become more stable, resembling that of an adult, even though changes in the endocrine, metabolic, dietary, and behavioral aspects continue to affect microbial function. The hormonal changes that occur at puberty are associated with alterations in various taxa, such as Bacteroides, Ruminococcus, and the butyrate-producing Lachnospiraceae [64]. Direct evidence linking adolescents’ gut microbiome to HPV vaccine immunogenicity remains limited. Although microbial communities that produce SCFAs have immunoregulatory properties that could, in theory, support vaccine responses, further longitudinal studies are still needed to link these microbial characteristics to the magnitude and duration of HPV vaccine antibody levels [65]. On the other hand, factors such as antibiotic use, a poor diet, psychosocial stress, and other disturbances may temporarily disrupt microbial metabolism and the inflammatory state [66,67,68,69]. They might thus alter the immunological environment in which either primary or booster vaccine responses are produced. More longitudinal studies are needed to determine whether these microbiota changes directly affect vaccine immunogenicity in adolescents or mainly reflect broader physiological and environmental differences.

Adulthood: In healthy adulthood, both the intestinal microbiota and the immune system are relatively stable and thus create a generally beneficial setting for strong vaccine responses. Nevertheless, microbial function stays responsive to diet, medications, infection, stress, and other environmental factors. Disrupting this stable ecosystem provides some of the strongest evidence that the microbiota can affect vaccine immunity. For instance, short-term administration of broad-spectrum antibiotics has been linked to reduced plasmablast responses and modified antibody responses after influenza vaccination [66]. These results demonstrate that disrupting microbial signaling and metabolic activity at the time of immunization can affect early innate and B-cell responses necessary for optimal humoral immunity.

In contrast, in some adult groups, greater microbial diversity and a higher presence of metabolically active commensal bacteria such as *Faecalibacterium*, *Roseburia*, and *Akkermansia* have been linked to stronger serological or cellular responses [21]. This may be because a well-functioning microbial ecosystem preserves epithelial integrity, controls systemic inflammation, and produces metabolites that support antigen-presenting cells and lymphocyte metabolism. The importance of this relationship is particularly notable in immunocompromised adults, since in them underlying diseases, chemotherapy, HIV infection, immunosuppressive therapy, and frequent exposure to antimicrobials can all occur together with both dysbiosis and impaired immunity [70,71]. In such populations, a decreased microbial metabolic capacity and increased inflammatory signaling may contribute to the inherent defects in antigen presentation and lymphocyte function, leading to weaker or less lasting vaccine responses.

Older Adults: Aging provides one of the clearest examples of how microbiome remodeling may converge with intrinsic immune dysfunction to compromise vaccination. Older age is frequently accompanied by reduced microbial diversity, depletion of beneficial or metabolically active commensals, expansion of opportunistic organisms, impaired epithelial barrier integrity, and altered microbial metabolite production [72]. These changes occur alongside immunosenescence and chronic low-grade inflammation (“inflammaging”), characterized by impaired antigen-presenting cell function, reduced naïve T-cell availability, altered T-cell responses, and diminished B-cell and germinal-center activity. Rather than acting independently, age-associated dysbiosis and immunosenescence may therefore reinforce one another.

The breakdown of barrier integrity and the increase in pro-inflammatory microbial communities might lead to greater systemic exposure to microbial products, which in turn maintains inflammatory signaling and could interfere with the coordinated shift from innate to adaptive immunity required for an effective vaccination response. Meanwhile, depleting organisms that produce SCFAs may reduce the metabolic and regulatory signals that normally help maintain epithelial homeostasis and support lymphocyte function. These processes may explain the reduced seroconversion and shorter protection duration commonly seen in older adults after receiving vaccines against influenza, pneumococcal disease, herpes zoster, and SARS-CoV-2 [73,74]. Consistent with this model, SCFA-producing bacteria have been linked to better antibody persistence and stronger T-cell recall in older people [21,75].

The fact that the microbiota changes with age might mean this change can be modified to enhance vaccine responses in older individuals. That said, the microbiota is not the only explanation for why vaccines become less effective as people grow older. To restore normal microbial function and to improve immunity in older people, researchers have suggested measures such as increasing dietary fiber, consuming fermented foods, or taking prebiotics, probiotics, synbiotics, and postbiotics [76,77,78,79]. Yet larger, more carefully controlled studies are needed to determine whether these methods can help reverse the age-related decline in vaccine effectiveness.

Age, the microbiota, and vaccination relate throughout the lifespan as a changing microbiota–barrier–immune axis rather than as a series of age-related microbial patterns. In infancy, the immaturity of microbiota occurs at the same time as the development of mucosal and adaptive immunity; in adolescence and adulthood, a relatively stable microbiota helps to maintain immune homeostasis but is still susceptible to disruption from the environment; and in older age, microbial dysbiosis can combine with barrier dysfunction, inflammaging, and immunosenescence. These age-related changes can affect antigen presentation, lymphocyte differentiation, antibody production, and immune memory formation, leading to differences in the immunogenicity and duration of vaccine responses over the course of life. Yet much of the mechanistic evidence remains associated or comes from experimental models. Longitudinal studies in humans that combine microbiome composition with microbial metabolomics, barrier biomarkers, cellular immune phenotyping, and vaccine-specific antibody and memory responses will be essential to distinguish causal microbial mechanisms from age-related associations and to identify microbiota-targeted approaches to improve vaccination outcomes.

## 4. Factors Modulating Gut–Vaccine Interactions

Vaccine responses occur within a gut microbial ecosystem that is constantly influenced by diet, medications, infections, environmental exposures, and socioeconomic conditions. These factors can affect immunogenicity by acting directly on the host’s immune system and by changing the structure of the microbial community, its functional potential, metabolite production, and the integrity of the intestinal barrier. Perturbations of the microbiome in this way may then alter the immune environment at the time of vaccination, including antigen-presenting cell activity, inflammation levels, lymphocyte activation, and antibody production or memory responses. Section 4 therefore views these exposures mainly as upstream regulators of the gut microbiome, providing a mechanistic link between environmental or clinical factors and variation in vaccine responsiveness from one individual to another (Table 1).

Nutrition as a Modulator of Microbial Function: Diet is among the strongest modifiable determinants of gut microbial ecology. Dietary fiber, resistant starch, and other fermentable carbohydrates promote saccharolytic microorganisms, including *Bifidobacterium*, *Roseburia*, and *Faecalibacterium*, and increase microbial SCFA production [79]. In contrast, diets dominated by refined carbohydrates and saturated fats can reduce microbial diversity, decrease beneficial metabolic functions, and favor expansion of pro-inflammatory or opportunistic organisms [97].

These diet-induced microbial changes may alter vaccine responsiveness indirectly by modifying epithelial barrier integrity, microbial metabolite availability, and basal inflammatory tone. For example, reduced abundance or activity of SCFA-producing communities may weaken metabolic and regulatory signals that normally support mucosal homeostasis and coordinated antigen-specific immune responses. Polyphenol-rich foods provide another microbiome-dependent influence because poorly absorbed dietary polyphenols can be transformed by intestinal microorganisms into bioactive metabolites with immunomodulatory properties [98,99].

Micronutrient deficiencies may similarly interact with microbial dysbiosis, particularly in populations experiencing malnutrition or environmental enteric dysfunction [100,101]. Thus, the association between nutritional status and vaccine immunogenicity should not be interpreted exclusively as a direct nutrient–immune interaction; altered microbial ecology and metabolic output may represent an important intermediate pathway. This relationship is particularly relevant to oral vaccines in infants and children living in resource-limited settings, where malnutrition, microbial immaturity, and intestinal inflammation frequently coexist [102].

Antibiotics and Other Medications as Microbiome Perturbations: Antibiotic exposure provides some of the clearest evidence that disruption of an established gut microbial community can modify vaccine responses. Broad-spectrum antibiotics can rapidly decrease microbial diversity, deplete metabolically important commensals, and alter microbial metabolic output. In experimental models, such perturbation has been associated with reduced plasmablast expansion, impaired germinal-center activity, and weaker antibody responses. At the same time, recolonization or supplementation with selected microbial metabolites can partially restore these defects [103,104,105].

Human studies provide complementary evidence. Antibiotic exposure around influenza vaccination has been associated with altered systemic metabolic and inflammatory responses and reduced antibody responses under selected conditions [106]. These observations support the concept that an intact microbial ecosystem may contribute to optimal vaccine responsiveness. However, the magnitude of this effect depends on baseline immunity, vaccine type, antimicrobial regimen, and host characteristics.

Non-antibiotic medications can also reshape the microbiome. Proton pump inhibitors and H2-receptor antagonists modify gastrointestinal pH and downstream microbial composition. At the same time, metformin, NSAIDs, and several psychotropic medications have been associated with distinct changes in microbial communities and metabolic pathways [107,108]. However, evidence directly linking these drug-induced microbiome alterations to vaccine outcomes remains limited. Consequently, these medications should be regarded as potential microbiome-mediated modifiers of vaccine responsiveness rather than established determinants of vaccine efficacy.

Infections and Co-infections as Drivers of Dysbiosis and Barrier Dysfunction: Enteric infections can disrupt the gut microbiome through loss of commensal organisms, expansion of opportunistic taxa, epithelial injury, and persistent intestinal inflammation. Recurrent infections involving organisms such as *Giardia*, *Cryptosporidium*, and enterotoxigenic *Escherichia coli* may contribute to environmental enteric dysfunction, characterized by impaired barrier integrity and altered mucosal immune homeostasis [109,110,111].

These changes are particularly relevant to oral vaccination because effective antigen sampling and mucosal immune activation depend on a relatively intact intestinal environment. Persistent dysbiosis and inflammation may interfere with antigen handling and the generation of coordinated mucosal antibody responses, providing one possible explanation for the reduced performance of rotavirus, oral polio, and cholera vaccines reported in populations with a high burden of enteric disease.

*Helminth* infection, HIV, tuberculosis, and other chronic infections can further modify both immune function and intestinal microbial ecology [112,113,114,115]. Their effects on vaccine responsiveness likely involve a combination of direct immune suppression or polarization and indirect microbiome-mediated mechanisms. Distinguishing these pathways remains important because infection-associated dysbiosis may represent a potentially modifiable component of impaired vaccine responsiveness.

Geography and Socioeconomic Environment as Ecological Modulators: Geographic differences in microbiota composition reflect combined influences of diet, sanitation, antimicrobial exposure, environmental microorganisms, infection burden, and lifestyle [116,117,118]. Rural and agrarian populations, for example, often harbor microbial communities enriched in fiber-adapted taxa such as Prevotella and Treponema. In contrast, Bacteroides-dominated communities are more frequently reported in industrialized settings [116]. These differences should not be interpreted as intrinsically beneficial or detrimental; rather, they show how strongly environmental context shapes microbial ecology.

Socioeconomic disadvantage can influence this ecosystem through overlapping exposures to malnutrition, unsafe water, recurrent enteric infection, antibiotic use, and poor sanitation. In children, these conditions can contribute to microbial immaturity, intestinal barrier dysfunction, and chronic mucosal inflammation, which may partly underlie geographic differences in responses to oral vaccines [87,88,119]. The relationship is therefore unlikely to follow a simple sequence in which geography itself determines vaccine efficacy. Instead, environmental and socioeconomic exposures converge on both the microbiome and the host immune system, producing population-specific immunological contexts in which vaccines are administered.

Gut Microbial Intervention: Probiotics, Prebiotics, Synbiotics, and Postbiotics: Researchers are investigating deliberate modulation of the microbiota as a potential way to modify the immunological environment surrounding vaccination. *Lactobacillus rhamnosus* GG, *Bifidobacterium breve*, and *Saccharomyces boulardii* have shown potential to increase IgA titers and seroconversion rates to oral vaccines, including rotavirus and cholera vaccines, and to improve antibody responses to parental influenza vaccines [89,90,91]. Effects are strain-specific and depend on timing relative to antigen exposure. Prebiotics such as galacto-oligosaccharides (GOS), inulin, and resistant starch are non-digestible substrates that selectively stimulate beneficial commensals and enhance SCFA production [92,93]. Prebiotics can modify microbial metabolic activity and epithelial barrier function; whether these effects translate into improved vaccine-specific immune memory remains incompletely established [120,121,122]. Synbiotics (which combine probiotics and prebiotics) are being evaluated in infants and children in low-income countries to improve oral vaccine performance [59,94]. Early trials report modest improvements in rotavirus and polio immunogenicity, although results vary. Postbiotics, including heat-killed bacteria, cell wall fragments, and purified microbial metabolites, offer an attractive option for immunocompromised populations where live organisms pose risks [95,96]. Experimental studies suggest that selected microbial metabolites and microbial-derived products can modulate dendritic-cell and lymphocyte functions relevant to adjuvant activity [123,124]. Rigorous, large-scale studies are needed to standardize dosing, strain selection, and delivery formats, and to confirm long-term safety and cost-effectiveness. In practice, the factors described above rarely act in isolation; they intersect to shape microbial diversity, metabolic output, and the immunological milieu at the time of vaccination. A malnourished child exposed to chronic enteric infections may also receive antibiotics, amplifying the risk of poor vaccine uptake [125,126,127]. Conversely, balanced diets, appropriate micronutrient status, and microbiome-supportive interventions can mitigate these risks and enhance the durability of protection.

These factors rarely operate independently. Diet can influence the microbiome’s resilience to antibiotic exposure; infection can alter nutritional status and epithelial integrity; socioeconomic conditions determine exposure to both pathogens and medications; and host age or disease can modify the consequences of each perturbation. Their combined effect is therefore more accurately represented as an interconnected ecological network than as a series of isolated vaccine determinants.

## 5. Microbiome–Vaccine Interactions: Converging Evidence from Human Cohorts and Mechanistic Studies

Studies involving human randomized interventions, observational cohorts, and experimental models (Table 2) have demonstrated the connection between the gut microbiota and vaccine responsiveness. While these evidence types are complementary, they should not be regarded as equivalent. Observational studies in humans show associations between characteristics of the microbiota and the results of vaccination; yet, in general, they are unable to tell whether these associations are due to causation or to confounding factors such as age, diet, the use of medication, geographical location, past infection, or the initial state of the immune system. Randomized interventions targeting the microbiota provide stronger evidence that altering the intestinal ecosystem can affect certain vaccine responses, although effects remain highly variable. Germ-free, antibiotic-treated, gnotobiotic, and humanized animal models provide useful mechanistic support but do not by themselves establish clinical relevance for humans. For this reason, this section focuses on evidence from human interventions and observational studies, with experimental models used mainly to interpret underlying mechanisms.

Observational Studies in Humans: Population-based studies in diverse settings show that microbial composition and function correlate with vaccine responsiveness. In Indian and Malawian infants, enrichment of *Bifidobacterium longum subsp. infants* and of other saccharolytic bacteria would be associated with higher seroconversion rates for the oral polio, BCG, and rotavirus vaccines [128]. In the United States, groups of individuals had higher abundance of Collinsella and greater microbiome stability during the first months of life, coupled with better rotavirus IgA titers [129]. In adults, baseline microbiota diversity and the presence of taxa that produce short-chain fatty acids (for example, *Faecalibacterium prausnitzii and Roseburia* spp.) correlate with stronger plasmablast responses to seasonal influenza vaccines [129,130]. Exposure to antibiotics before and during immunization is repeatedly linked to lower antibody titers and delayed generation of memory B cells, highlighting the importance of an intact microbial community at the time of vaccination [131]. In older adults, the continued presence of butyrate-producing organisms and the overall ecological resilience predict a sustained response to both influenza and pneumococcal vaccines [132,133]. On the other hand, frailty, the presence of multiple chronic conditions, and ‘inflammaging’ are often seen together with dysbiosis and are linked to worse serological outcomes.

Randomized Controlled Trials with Microbiota-Targeted Interventions: Interventional studies provide more direct evidence regarding causality. Several randomized controlled trials (RCTs) have examined the effect of administering probiotics or synbiotics either before or during vaccination [79,120,121]. Clinical intervention studies indicate that certain microbiota-targeted interventions can alter vaccine-induced immune response. Yet the size and consistency of these effects vary by strain, vaccine, dose, population, and study design. In infants, daily supplementation with probiotic strains such as *Lactobacillus rhamnosus GG* and *Bifidobacterium breve* has been found to enhance mucosal IgA responses and increase seroconversion rates for oral rotavirus and cholera vaccines. However, the extent of benefit varies by strain, dose, and population [59,122,134]. In adults, consumption of probiotic preparations or fermented dairy products containing *Lactobacillus casei Shirota* has been linked to significantly higher antibody titers after receiving an influenza vaccine given by parenteral route, indicating improved systemic humoral immunity [135,136]. Building on these results, randomized trials of synbiotics that combine fermentable prebiotics (for example, GOS) with specific probiotic strains in South Asian populations have reported small but consistently observable improvements in the immunogenicity of the oral polio vaccine [143,144,145]. Together, these studies provide interventional evidence that certain microbiota-directed approaches can modify vaccine-related immune responses under specific experimental and clinical conditions. Nevertheless, the magnitude and reproducibility of these effects depend on the microbial strain or formulation, the vaccine platform, the study population, the baseline microbiota, and the immune endpoint; thus, they have not yet established a generally applicable causal benefit of microbiome modulation across different vaccines or populations.

Antibiotic Perturbation as a Human Mechanistic Probe: Among human studies, antibiotic perturbation is particularly informative because it allows investigators to examine vaccine responses following a deliberate disturbance of the intestinal microbiota. Because broad-spectrum antibiotics can quickly reduce microbial diversity and alter microbial metabolic activity, they provide a suitable experimental approach for determining whether the loss of microbial functions affects vaccine-induced immunity.

Studies in the context of influenza vaccination have shown that antibiotic exposure can alter systemic metabolic profiles, inflammatory signaling, and certain antibody responses [131]. This is important because it goes beyond simple cross-sectional associations and suggests that a temporary disruption of the intestinal microbial ecosystem can affect the physiological environment associated with vaccination.

Yet antibiotic studies also have important limitations: different drug classes affect microbial communities in different ways, antibiotics may affect vaccine responses independently of the microbiome, and vaccine responses are strongly influenced by pre-existing immunity. While these experiments offer stronger evidence for a role of the microbiome than do conventional observational studies, they still do not identify a single microbial organism or metabolite as the causal mediator.

Experimental Models as Mechanistic Supporting Evidence: Experimental models provide mechanistic context for the associations observed in humans but should be interpreted as supportive rather than clinically equivalent evidence. Germ-free and antibiotic-treated mice frequently exhibit impaired antibody responses, reduced plasmablast expansion, or altered germinal-center reactions following immunization [137,138,139]. Restoring microbial communities or specific microbial signals may recover some of these defects, supporting a mechanistic role for commensal-derived signals in immune priming.

Gnotobiotic models also enable the testing of defined microbial communities under controlled conditions. Studies of this type show that microbial metabolites, such as SCFAs, can affect B-cell differentiation, class-switch recombination, and the development of memory T-cells [140,141,142]. Humanized animals colonized with microbiota from human donors can likewise reproduce certain donor-associated immune phenotypes, linking clinical observations with mechanistic testing [149].

These models are useful because they allow manipulations that could not be carried out in humans. Nevertheless, due to germ-free physiology, simplified microbial communities, controlled diets, and differences in the immune system among species, it is not possible to directly apply the findings to human vaccination. Their main value therefore lies in identifying potential mechanisms which can then be examined in well-designed human studies, not in proving the clinical efficacy of a vaccine.

## 6. Host–Microbe–Vaccine Interactions Beyond the Gut

**Figure 1:**
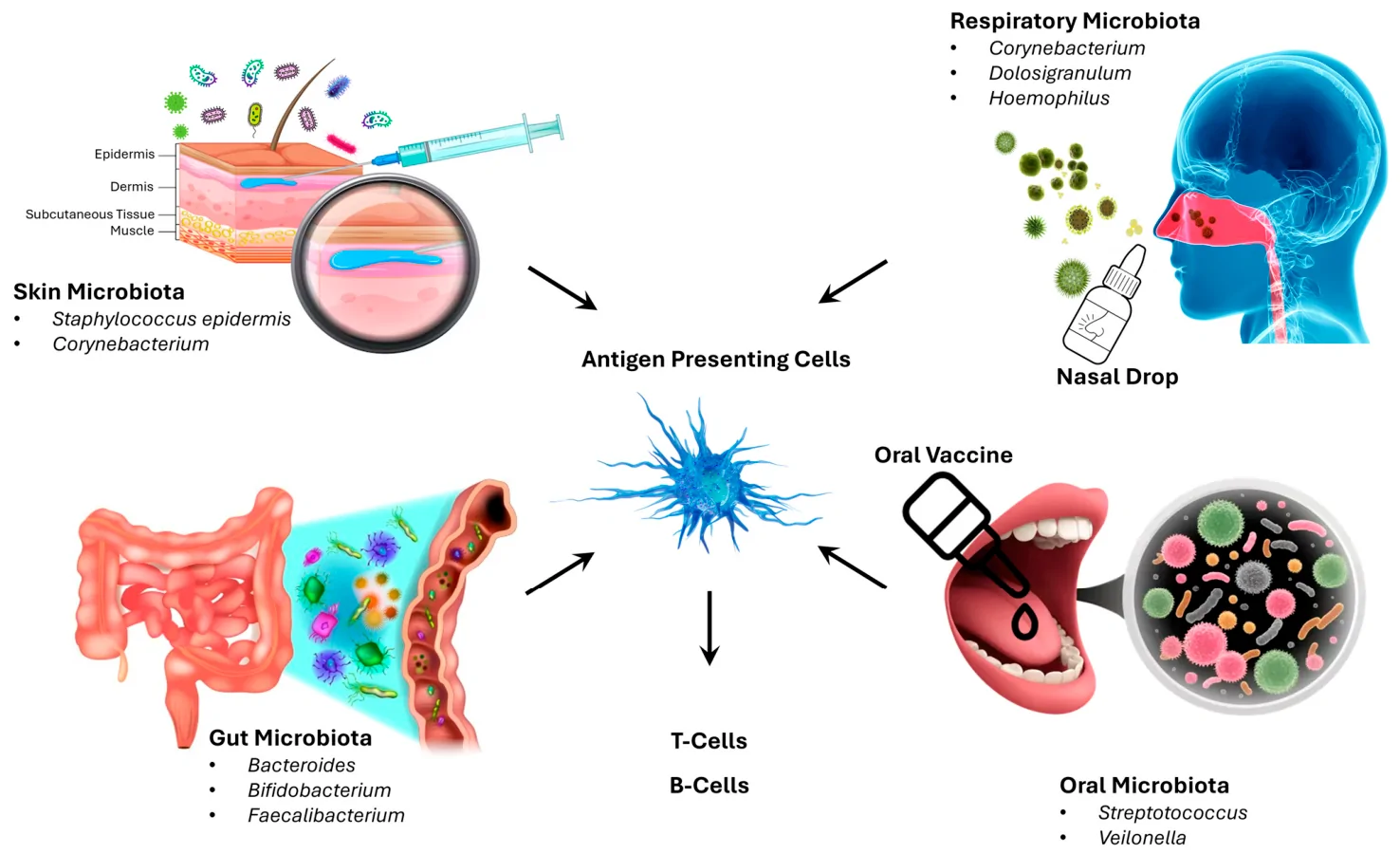
The regulation of vaccine-induced immunity by microbiota at multiple sites. The figure shows how microbiota at various sites in the body, such as the skin, respiratory tract, gut, and oral cavity, help shape the host’s immune response to vaccination. On the skin, commensal microorganisms like *Staphylococcus epidermidis*, *Cutibacterium acnes*, and *Corynebacterium* spp. interact with keratinocytes and antigen-presenting cells to control local inflammation and promote immune activation after intradermal or transcutaneous vaccination. In the respiratory tract, the nasal microbiota, which includes organisms such as *Corynebacterium*, *Dolosigranulum*, and *Haemophilus*, affects epithelial signaling, type I interferon responses, and mucosal IgA production, thereby impacting the effectiveness of intranasal vaccines. In the gut, a diverse microbial community (for example, *Bacteroides*, *Faecalibacterium*, and other taxa that produce short-chain fatty acids) controls systemic immune priming by regulating dendritic cell activation, T-cell differentiation, and germinal center responses following vaccination. The oral microbiome provides early antigen exposure and promotes immune priming by altering interactions in the oropharyngeal mucosa, thereby influencing antigen uptake and mucosal immunity. Signals from these different microbial habitats are directed to antigen-presenting cells (APCs), activating adaptive immune pathways such as T-cell polarization and B-cell maturation. This integrated network shows how microbiota at different anatomical locations influence vaccine responsiveness, underscoring the need for a systems-level, whole-body approach in vaccinology. Abbreviations: APCs, Antigen-Presenting Cells; IgA, Immunoglobulin A; SCFAs, Short-Chain Fatty Acids.

Although most research on microbiota and vaccination has focused on the intestinal tract, commensal ecosystems at other barrier sites also affect immune function and may influence vaccine effectiveness (Figure 1). The skin, the respiratory mucosa, and the oral cavity each contain dense populations of actively metabolizing organisms which interact with both local and systemic immunity, thereby adding further layers of regulation to vaccine-induced protection.

Skin Microbiota and Cutaneous Vaccination: The skin is both an immunological sentinel and a reservoir of diverse microorganisms, including *Staphylococcus* (S.) *epidermidis*, *Cutibacterium acnes*, *Corynebacteria*, and various fungi [150,151,152]. These microbes influence keratinocyte signaling, antimicrobial peptide production, and maturation of skin-resident DCs and Langerhans cells [153,154,155]. They modulate the balance between tolerogenic and pro-inflammatory responses, thereby conditioning the environment in which intradermal vaccines operate [156]. Experimental data suggest that commensal S. epidermidis can enhance IL-1-dependent activation of dermal T cells and support the generation of tissue-resident memory cells after skin challenge [157]. In murine models, antibiotic depletion of the cutaneous flora impairs responses to intradermally delivered vaccinia or inactivated influenza, whereas recolonization restores antigen-specific T-cell priming [158,159]. Human studies with intradermal BCG and novel microneedle platforms indicate that baseline skin microbiota diversity may correlate with the magnitude of Th1 responses, though causal links remain to be established.

Respiratory Microbiota and Intranasal Vaccines: The upper respiratory tract harbors a complex microbial network, including *Corynebacterium*, *Dolosigranulum*, *Staphylococcus*, and non-typeable *Haemophilus* [160,161]. These microbes help shape mucosal immunity by activating epithelial PRRs and altering type I and III interferon pathways. They also affect secretory IgA production and the development of tissue-resident memory T cells, both of which contribute to protection against airborne pathogens like SARS-CoV-2 Infection [160,161,162]. Evidence from intranasal influenza vaccination indicates that the makeup of the nasopharyngeal microbiota can influence seroconversion and local antibody levels [12,163]. In children, those whose microbiota is dominated by *Dolosigranulum* or *Corynebacterium* show more robust mucosal IgA responses than those who have a higher presence of *Moraxella* or *Streptococcus pneumoniae* [12,163,164]. In adults, prior antibiotic exposure before receiving an intranasal vaccine decreases commensal Corynebacterium abundance and is linked to reduced plasmablast expansion [12]. Similarly, studies involving live-attenuated RSV and pertussis vaccines are beginning to show similar dependencies, implying that respiratory commensals might function as natural adjuvants or as gatekeepers of mucosal tolerance [165].

Oral Microbiome and Uptake of Oral Vaccines: The oral cavity contains diverse communities in the tongue, on the buccal mucosa, in the gingiva, and within the tonsillar crypts. The main types of microbes include *Streptococcus*, *Veillonella*, *Neisseria*, and anaerobes such as *Prevotella*. These microorganisms generate metabolites, help to preserve the integrity of the epithelium, and play a role in training the local immune cells in Waldeyer’s ring (that is, in the palatine and adenoidal tonsils), which is a key area for the induction of mucosal immunity [166]. For oral vaccines such as those for polio, cholera, and rotavirus, the antigen first contacts the oropharyngeal mucosa before reaching the gut. In the gut, the commensal bacteria might affect antigen stability, M-cell uptake, and dendritic cell presentation. Dysbiosis from poor oral hygiene, a high-sugar diet, or frequent use of antibiotic rinses might interfere with these processes and could therefore reduce IgA titers or alter T-cell polarization [166,167]. On the other hand, keeping the oral microbiota in balance could help to improve the initial phases of mucosal immune interaction. Overall, recent studies in the skin, respiratory system, and oral cavity show that vaccine effectiveness results from a network of interactions between microbes and the immune system throughout the body. Although each surface has its own taxonomic communities, metabolites, and immune structures, they all share the same fundamental principles concerning barrier integrity, PRR signaling, and communication from the local to the systemic level. Including these ecosystems in vaccinology research, especially when developing mucosal or needle-free vaccines, might lead to the discovery of new adjuvant approaches and biomarkers of response beyond the gut.

## 7. Microbiota and Different Vaccine Platforms

The effect of the gut microbiota on vaccination depends on the immunization method. Various types of live attenuated vaccines, inactivated vaccines, subunit formulations, nucleic acid-based technologies, and adjuvanted delivery systems interact with the immune system via different pathways and to different extents, depending on the host’s microbial ecosystem [5,168]. Understanding how commensal bacteria influence responses to these platforms provides insight into variability in efficacy and opens possibilities for improving design and implementation, as shown in Table 3.

Live Attenuated Vaccines (LAVs): LAVs contain weakened pathogens that replicate only partially in the host. Because they mimic natural infection, they elicit robust, durable immunity, often with a single dose [169]. Their performance, however, is particularly sensitive to the state of the mucosal environment and the microbiota. Oral vaccines such as those for rotavirus, poliovirus (OPV), cholera, and typhoid need to colonize or replicate in the intestinal lumen to initiate both mucosal and systemic immunity [169]. Research in South Asia and sub-Saharan Africa has shown that infants with high levels of *Bifidobacterium longum subsp. infantis* exhibit better seroconversion to both rotavirus and OPV [170]. In contrast, increased numbers of Enterobacteriaceae, Clostridioides difficile, or other pathobionts are linked to lower antibody titers and delayed IgA responses. Environmental enteropathy, marked by chronic gut inflammation and disruption of the gut barrier, can also reduce vaccine uptake [110,111]. Similarly, intranasal LAVs, such as the live attenuated influenza vaccine (LAIV), rely on local commensal bacteria. A dominant presence of *Corynebacterium* and *Dolosigranulum* in the nasopharynx is linked to a more robust mucosal IgA response, while an increase in Moraxella or Streptococcus pneumoniae may inhibit the response, perhaps by changing the interferon tone or by affecting antigen uptake [12,163,164]. These findings show that the effectiveness of LAVs is closely related to the quality of the microbial and epithelial environment at the site of administration.

Inactivated and Subunit Vaccines: Inactivated (killed) vaccines and protein- or polysaccharide-subunit formulations lack replicative capacity and rely on direct uptake by APCs and on adjuvant signals to induce immunity. Traditionally, they have been regarded as less dependent on gut ecology, yet emerging data suggest that microbial composition can still affect their immunogenicity. For parenteral vaccines such as inactivated influenza, tetanus toxoid, diphtheria, and hepatitis B vaccines, the microbiota’s impact seems to work mainly by affecting baseline immune function. In adults with low baseline immunity, taking antibiotics before receiving an influenza vaccine led to reduced IgG1 and IgA binding and neutralization responses [21,171]. Observational studies have found that a greater abundance of bacteria that produce SCFAs (*Faecalibacterium*, *Roseburia*, *Bifidobacterium*) is associated with more prolonged antibody persistence [21,171]. SCFAs and other microbial metabolites might improve the metabolic fitness of long-lived plasma cells and promote the differentiation of T-follicular helper cells, both of which are important for the long-term serological response [21]. Conjugate vaccines for *Haemophilus influenzae* type b or pneumococcus also show some sensitivity to the microbiota, possibly because polysaccharide antigens require strong T-cell help to elicit an optimal response [173]. However, compared with LAVs, the microbiota’s influence is smaller, indicating reduced dependence on mucosal replication.

mRNA Vaccines: Messenger RNA vaccines mark a significant advance in vaccinology, as shown by the rapid development of COVID-19 vaccines. They consist of lipid-encapsulated RNA that encodes antigen proteins, which host cells then translate to produce both humoral and cellular immunity [173]. Since these vaccines avoid the need for microbial replication, it might be thought that the microbiota has little effect. However, evidence shows that signals from commensal microbes can contribute to the baseline immune set point and thus affect the quality of the subsequent immune response. Mice without microbiota showed reduced activation of antigen-presenting cells after receiving an mRNA vaccine, while the presence of microbes was linked to increased CD8+ T cells in response to vaccination [174]. In humans, reduced microbiota diversity during COVID-19 mRNA vaccine administration is associated with lower neutralizing antibody titers and short-lived plasmablast responses [18,174]. SCFAs, tryptophan catabolites, and secondary bile acids may drive these effects by influencing dendritic cell maturation and regulating inflammatory thresholds during antigen presentation [9]. Furthermore, microbiota-derived products can work together with the innate sensing mechanisms activated by the RNA platform (for example, TLR7/8, RIG-I) [40]. A balanced commensal microbiome can therefore enhance interferon-mediated adjuvant effects without causing excessive reactogenicity.

Viral-Vector Vaccines: Recombinant viral vectors (for example, adenovirus or the modified vaccine Ankara) introduce genes that code for antigens into host cells. The effectiveness of such vaccines relies on efficient transduction and activation of innate immune responses through nucleic acid sensors. The microbiota may indirectly influence these processes by affecting baseline interferon pathways and the number of tissue-resident antigen-presenting cells. Analysis of adenovirus-based COVID-19 vaccine trials found that people with a more diverse microbiota and higher levels of butyrate-producing bacteria had more prolonged spike-specific antibodies and memory T cells [171]. If dysbiosis or depletion of the microbiota occurs before receiving an adenoviral vaccine, it reduces early inflammatory cytokines and hinders CD8+ T cell expansion; this effect can be reversed by re-establishing the microbiota with commensal microbial communities.

Adjuvants and Delivery Systems: Adjuvants are designed to enhance immunogenicity; their effectiveness is influenced by the surrounding immune environment, particularly by microbial products [39]. Alum, a long-used adjuvant, causes local cell death and releases danger-associated molecules. Previous microbial exposure can affect its ability to promote Th2-skewed responses by programming pathways in macrophages and dendritic cells [176]. MF95 and other squalene-based emulsions attract monocytes and neutrophils to the injection site; SCFAs and other microbial metabolites can influence chemokine gradients and antigen-presenting cell metabolism, thereby affecting antibody response kinetics [177]. TLR agonists such as CpG (a ligand for TLR9) or poly(I:C) (a ligand for TLR3) mimic microbial ligands; their downstream effectiveness can increase or decrease depending on the level of baseline activation of the host’s PRRs by commensal organisms [178]. Delivery innovations, such as microneedles, lipid nanoparticles, and mucosal sprays, also interact with the local microbiota by changing tissue permeability or by coming into close association with commensal microbes that function as natural adjuvants. Available evidence indicates that the degree of interaction between the microbiota and the immune system may vary across vaccine platforms, although the clinical significance of these relationships is not yet fully understood. Vaccines based on live-attenuated organisms are most susceptible to disruption of the mucosal ecosystem, while mRNA and vector-based vaccines involve microbiota-modulated innate pathways that regulate the magnitude and duration of the response. Adjuvants and delivery systems also incorporate microbial signals. Considering these interactions will help select a platform, design adjuvants, and develop strategies that support microbiota to improve immunization across different populations and life stages.

## 8. Microbiota–Vaccine Interactions in Special Populations

Vaccine reactions differ greatly among population groups because of differences in host immunity, comorbid conditions, and the ecological state of the microbiota. To design immunization strategies that provide fair protection, it is essential to understand these different contexts. Four groups illustrate the relationship between microbial ecology and vaccine effectiveness: infants born prematurely and those with low birth weight, pregnant and lactating women, immunocompromised individuals, and people who have chronic inflammatory or metabolic diseases (Table 4).

Preterm Infants and Low-birthweight Neonates: Premature birth and intrauterine growth restriction profoundly influence early microbial colonization and immune maturation. Preterm infants acquire fewer maternal vaginal and fecal microbes at birth and often require neonatal intensive care, antibiotics, and formula feeding, which delay the establishment of *Bifidobacterium* and other saccharolytic taxa [179,180]. Their microbiota is typically enriched in facultative anaerobes (e.g., *Enterobacteriaceae*, *Staphylococcus*), and SCFA concentrations are markedly reduced [179,208]. This ecological immaturity parallels deficits in innate and adaptive immunity, including reduced TLR signaling, impaired dendritic-cell priming, diminished T follicular helper cells, and weaker germinal center formation. Consequently, preterm neonates show attenuated seroconversion to vaccines such as hepatitis B and Hib, as well as lower antibody persistence to Hib and pneumococcal conjugates [209,210,211]. Strategies to address these vulnerabilities include encouraging maternal milk feeding, which provides Bifidobacterium and oligosaccharides, limiting unnecessary antibiotic use, and considering targeted probiotics or synbiotics to accelerate microbial maturation [212,213]. Adjusting the timing or number of doses, such as an early two-dose priming schedule for hepatitis B, may also compensate for immature immunity.

Pregnant and Lactating Women: Pregnancy induces physiological remodeling of the microbiota, with enrichment of Proteobacteria and Actinobacteria and a shift in metabolic output [182,214]. These changes are thought to support energy harvest and immune tolerance but may also influence vaccine immunogenicity. Maternal immunization with Tdap, influenza, or COVID-19 vaccines elicits robust antibody responses, yet titers can vary with maternal nutritional status and gut microbial composition [185]. Microbiota can also mediate vertical effects on the fetus and infants [215,216]. SCFAs, vitamins, and microbial cell-wall components cross the placenta, shaping fetal immune development and potentially “training” innate cells to respond to vaccines after birth [51,191]. Breast milk provides additional cues: it contains secretory IgA, antimicrobial peptides, human milk oligosaccharides, and live commensals that help seed the infant gut and calibrate mucosal immunity [184,185]. Perturbations of maternal ecology, such as obesity, gestational diabetes, excessive hygiene, or peripartum antibiotics, may dampen maternal vaccine responses and alter the transfer of protective antibodies [217,218]. Pilot studies suggest that maternal supplementation with specific probiotics or fiber-rich diets can enhance vaccine-induced antibody titers and increase breast milk IgA levels, though the evidence is preliminary.

Immunocompromised Hosts: People with impaired immunity make up a diverse but highly important group for vaccination, since gut microbiota disturbances are common and closely linked to reduced immunogenicity. Those who have received solid-organ or hematopoietic stem-cell transplants are subjected to long-term immunosuppressive treatments and broad-spectrum antibiotics, which greatly alter the intestinal ecosystem by usually depleting the obligate anaerobes and the types of bacteria that produce short-chain fatty acids (SCFAs), while at the same time allowing opportunistic bacteria and fungi such as *Enterococcus* and *Candida* to increase in number [188,189,218,219]. This dysbiotic state hampers dendritic cell maturation, antigen presentation, and support for T follicular helper cells, reducing germinal center reactions and limiting serological responses to vaccines for influenza, hepatitis B, and SARS-CoV-2. Measures intended to restore microbial diversity and metabolic activity, such as enriching the diet with fiber, supplementing with targeted SCFAs, or using carefully chosen next-generation probiotics, could help improve the body’s response to vaccines. However, thorough safety assessments are necessary given the severe immunosuppression involved. HIV-positive individuals also show typical dysbiosis characterized by a loss of butyrate-producing *Clostridia*, an increase in Proteobacteria, and persistent disruption of the intestinal barrier [190,191]. As a result, microbial translocation, chronic immune activation, and defective germinal center activity continue even with effective antiretroviral therapy, leading to suboptimal antibody levels and poor memory formation following vaccination against HPV, hepatitis B, and pneumococcus [192,193,194,195,196,197,198,199,200,201,202,203,204,205,206,207,208,209,210,211,212,213,214,215,216,217,218,219,220]. Therapeutic methods that help to strengthen the epithelial layer and reestablish communities capable of producing SCFAs, for example, by adding prebiotic fiber, are currently being investigated in carefully controlled research studies. A varied microbial community may contribute to systemic immune priming by affecting dendritic cell activation, T-cell differentiation, and germinal center responses [191]. Patients undergoing treatment with biologics or targeted immunomodulators, including inhibitors of TNFα or IL-17 signaling, also experience changes in their microbial composition and metabolism because of therapy; these changes interact with microbiota-dependent immune conditioning [192]. Although vaccination is generally safe in such individuals, attenuated humoral responses to the influenza and COVID-19 vaccines have been reported, indicating that pharmacological modification of cytokine networks may impact vaccine effectiveness. Understanding how specific immunosuppressive pathways interact with metabolites derived from commensal microbes and PRR signaling could help inform rational choices about adjuvants, optimize booster schedules, and develop microbiota-informed interventions to maximize protective immunity in this susceptible group.

Individuals with Chronic Inflammation and Metabolic Diseases: Individuals with chronic inflammatory or metabolic diseases are a population in which vaccine responsiveness is frequently altered, in part due to sustained perturbations in the gut microbiota and in immune–metabolic balance. Obesity and type 2 diabetes are always linked with reduced microbial diversity, a depletion of commensal bacteria that produce short-chain fatty acids (SCFAs), and an increase in Gram-negative bacteria that produce LPS such as those in the family Enterobacteriaceae; this situation leads to systemic low-grade inflammation (“metaflammation”), damages the integrity of the barrier, and disrupts the immunometabolic pathways [193,194,195]. The pro-inflammatory and metabolically restricted environment thus hampers oxidative phosphorylation in dendritic cells and their glycolytic reprogramming, limits B-cell class switching and the differentiation of plasma cells, and reduces both the generation and maintenance of antigen-specific T-cell memory, offering a mechanism to explain the lower seroconversion rates and the faster waning of antibodies seen after influenza, hepatitis B, and SARS-CoV-2 vaccination in obese or insulin-resistant adults [221,222]. Inflammatory bowel disease (IBD) introduces an extra level of complexity by combining intrinsic dysregulation of the mucosal immune system with a dysbiotic microbiota characterized by the loss of anti-inflammatory species such as Faecalibacterium prausnitzii and the expansion of Proteobacteria, both of which impair the integrity of the epithelial barrier, antigen sampling, and the cytokine environment needed for proper germinal-center reactions [206,207]. Also, the immunosuppressive treatments used concurrently, such as corticosteroids and anti-TNF biologics, can further suppress the B- and T-cell responses triggered by vaccination, although most inactivated and subunit vaccines are still safe and remain largely immunogenic when given during periods of disease quiescence or before beginning treatment with potent biologic agents [223,224]. In all these chronic conditions, approaches that help to restore immunometabolic and microbial balance, such as enriching the diet with fermentable fiber and polyphenols to boost SCFA production, achieving structured weight loss to reduce metaflammation, and using antibiotics or acid-suppressing medications with care, are becoming increasingly recognized as helpful measures to improve vaccine effectiveness [225,226]. Interventions that target the microbiota, including synbiotics, defined postbiotics, and carefully selected FMT, also hold additional promise for adjusting the immune response in mucosal and systemic tissues, but their incorporation into vaccination programs will need to be based on the results of well-designed clinical trials to confirm their efficacy, durability, and safety in people who are both metabolically and immunologically vulnerable [147]. The interactions between the microbiota and vaccines extend beyond healthy individuals; in preterm infants, pregnant women, immunocompromised people, and patients with chronic inflammatory or metabolic disorders, immature, disrupted, or chronically inflamed microbial ecosystems can weaken both serological and cellular responses. By acknowledging these connections, targeted nutritional, microbial, and immunological interventions can be implemented to ensure all groups benefit fully from vaccination.

## 9. Advances in Technology and Methodology in the Study of Gut–Vaccine Crosstalk

**Figure 2:**
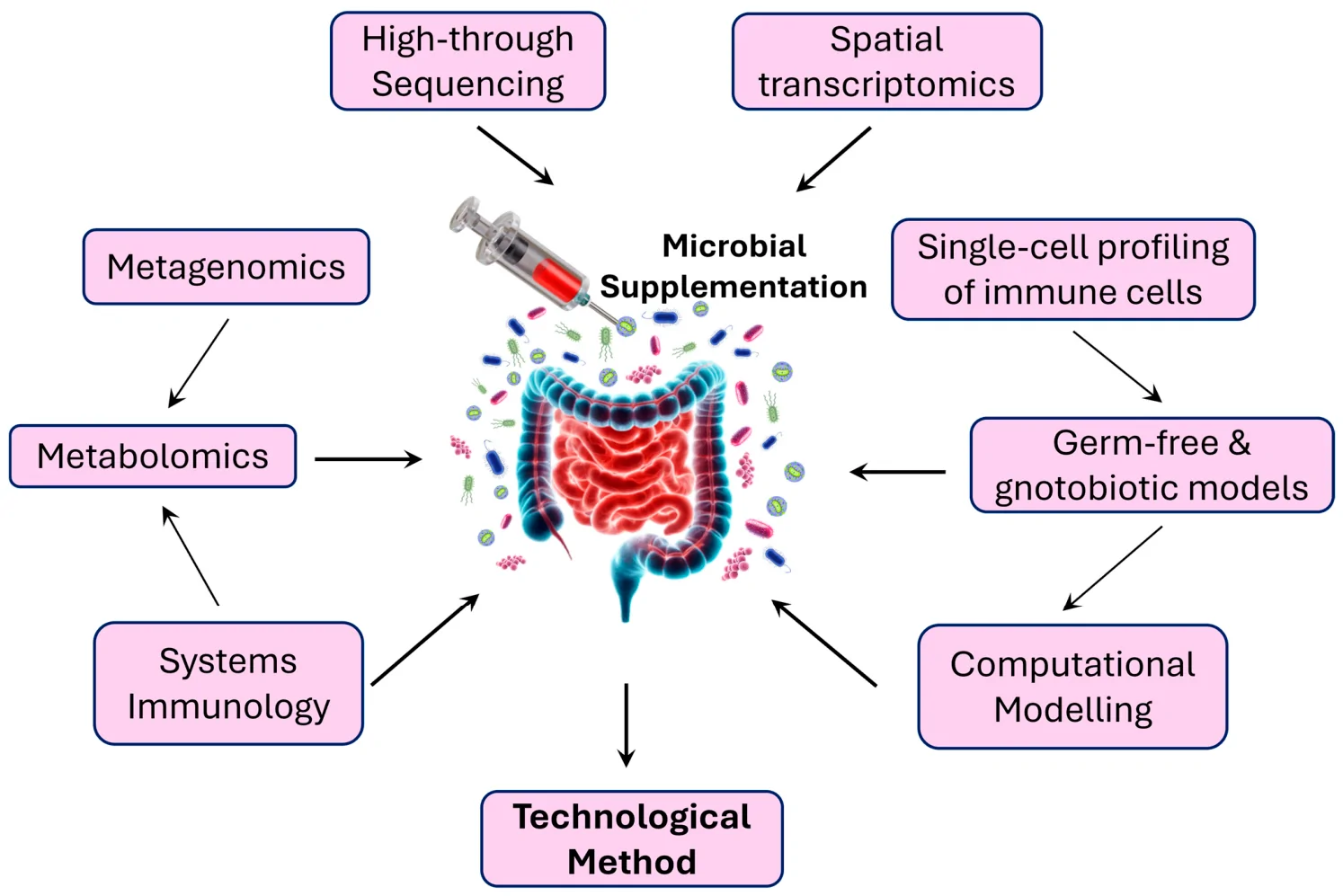
Framework for the study of interactions between the microbiota and vaccines. The figure shows the integrated use of multi-omics and experimental methods for examining microbiota–vaccine crosstalk at the molecular, cellular, and organismal levels. High-throughput sequencing techniques such as shotgun metagenomics and 16S rRNA sequencing enable taxonomic and functional analysis of the microbial communities associated with a person’s response to a vaccine. Functional omics components, such as metatranscriptomics, metaproteomics, and metabolomics, offer insights into microbial activity and how metabolites modulate the immune system, including short-chain fatty acids, bile acids, and tryptophan-derived metabolites. Advanced spatial and single-cell technologies, such as spatial transcriptomics, single-cell RNA sequencing (scRNA-seq), and ATAC-seq, enable high-resolution mapping of immune cell states, chromatin accessibility, and tissue-specific interactions between the host and microbes in gut-associated lymphoid tissue (GALT) and Peyer’s patches. Immune phenotyping techniques, such as high-dimensional cytometry (CyTOF and spectral flow cytometry), further enable characterization of cellular heterogeneity and vaccine-induced immune signatures. Experimental validation is obtained using culturomics, organoid and gut-on-a-chip systems, and germ-free or gnotobiotic animal models, which enable causal testing of the effects of microbiota on antigen presentation, barrier integrity, and adaptive immunity. Humanized mouse models act as a translational link by reproducing human immune responses in vivo. Lastly, computational methods, such as machine learning, network modeling, and digital twin simulations, integrate multi-omics datasets to predict vaccine responsiveness, identify microbial biomarkers, and help develop precision immunization strategies. Together, these complementary technologies form a systems-level approach for understanding and manipulating the microbiota’s regulation of vaccine efficacy. Abbreviations: 16S rRNA, 16S Ribosomal Ribonucleic Acid; ATAC-seq, Assay for Transposase-Accessible Chromatin using sequencing; CyTOF, Cytometry by Time-of-Flight; GALT, Gut-Associated Lymphoid Tissue; MALDI-TOF, Matrix-Assisted Laser Desorption/Ionization-Time of Flight; NMR, Nuclear Magnetic Resonance; RNA-seq (scRNA-seq), Single-cell Ribonucleic Acid Sequencing; SCFAs, Short-Chain Fatty Acids.

Rapid progress in analytical technologies has transformed understanding of how the intestinal microbiota shapes vaccine-induced immunity. Methods spanning sequencing, metabolomics, spatial and single-cell profiling, and computational systems biology now allow researchers to map the tripartite relationship among microbes, the mucosal barrier, and host immune cells at unprecedented depth (Figure 2). These approaches are shifting the field from descriptive correlations to mechanistic and predictive science.

High-Throughput Sequencing and Metagenomics: Next-generation sequencing (NGS) remains the foundation for profiling microbiota–vaccine interactions. Shotgun metagenomics provides strain-level resolution of bacterial, archaeal, viral, and fungal genomes, enabling the discovery of taxa associated with vaccine responsiveness [5,6]. Functional annotations of the metagenomic reads show metabolic pathways, such as butyrate synthesis, tryptophan catabolism, and bile acid biotransformation, which may affect dendritic cell priming or B cell class switching [227]. When metagenome-assembled genomes (MAGs) are assembled, previously uncultured organisms can be recovered, thus increasing the number of potential adjuvant species [228].16S rRNA sequencing is still useful when working with large groups of samples because of the cost or the number of samples, which make deep metagenomics impractical, although the taxonomic resolution it provides is limited [228,229]. Long-read sequencing (for example, using Oxford Nanopore or PacBio) is now employed to resolve repetitive regions and plasmids, making it possible to clarify the horizontal gene transfer of molecules with adjuvant-like properties (for example, flagellin and polysaccharide operons) [230]. When viromics and mycobiome profiling are integrated, it becomes clear that bacteriophages, eukaryotic viruses, and fungi also play a role in the immunological environment and thus influence antibody kinetics and mucosal tolerance [231].

Metatranscriptomics, Metaproteomics, and Metabolomics: Static gene inventories alone are insufficient to capture the functional state of microbial communities during the dynamic process of vaccination, necessitating multi-omics approaches that capture real-time activity at the transcript, protein, and metabolite levels. Metatranscriptomics allows for the quantitative analysis of RNA expression across the entire microbial community and thus shows the quick changes that take place in metabolic fluxes, stress-response systems, and biosynthetic pathways after exposure to an antigen [232,233], and it also makes it possible to identify the particular enzymes that are responsible for producing immunomodulatory molecules such as SCFAs and indole derivatives at the important stage of immune priming. Metaproteomics adds to these findings by directly recording microbial proteins expressed in vivo, including transporters, secreted effectors, and components of outer-membrane vesicles that can interact with PRRs and influence innate signaling pathways [234]. At the host–microbe interface, metabolomics carried out using mass spectrometry or nuclear magnetic resonance provides a comprehensive chemical picture of this interaction by detecting hundreds of small molecules that act as key immune modulators [235]. Untargeted techniques identify global metabolic profiles that are associated with vaccine responsiveness. In contrast, targeted assays accurately measure specific metabolites and link their concentrations to seroconversion rates, antibody durability, and T-cell memory strength. When combined with stable-isotope tracing, metabolomics also allows tracking nutrient flow from dietary substrates through microbial transformation to host tissues, demonstrating causal connections between nutrition, microbiota-derived metabolism, and the quality and longevity of vaccine-induced immunity [236,237].

Spatial Transcriptomics and Tissue Imaging: Spatially resolved technologies now enable direct visualization of how microbial signals interact with immune circuits across whole tissues, providing both anatomical and molecular context for vaccination-induced reprogramming. By using barcoded arrays or in situ sequencing, spatial transcriptomics records the expression of host genes across the intestinal villi, Peyer’s patches, and the draining lymph nodes, showing how vaccination alters epithelial cells, dendritic cells (DCs), and the stromal microenvironments in the presence of different microbial communities [238]. Imaging mass cytometry and multiplexed immunofluorescence also add a high-dimensional proteomic component by simultaneously measuring dozens of immune and structural markers, enabling reconstruction of antigen capture, antigen-presenting cell migration, and B-cell follicle organization at the single-cell level [239,240]. When combined with methods for localizing microbes, such as 16S rRNA fluorescence in situ hybridization or whole-genome probe detection, these techniques identify micro-niches where commensal bacteria, vaccine antigens, and host cells are found together, thereby locating the exact tissue sites where innate sensing and adaptive priming begin [241].

Single-Cell Profiling of Immune Cells: Single-cell technologies dissect how individual immune cells integrate microbial and vaccine-derived signals, uncovering heterogeneity that is obscured in bulk analyses. Single-cell RNA sequencing resolves transcriptional states of DCs, macrophages, innate lymphoid cells, and T- and B-cell subsets during vaccine responses. When integrated with paired TCR and BCR repertoire sequencing, it enables tracking of clonal expansion and differentiation of antigen-specific lymphocytes as a function of baseline microbiota composition [242,243,244]. Single-cell assay for transposase-accessible chromatin using sequencing (ATAC-seq) complements this by mapping chromatin accessibility landscapes, revealing how microbial metabolites such as butyrate or secondary bile acids imprint epigenetic programs that bias plasma blast longevity, T follicular helper differentiation, or regulatory T-cell stability [245]. High-parameter flow cytometry and mass cytometry (CyTOF) extend these insights to protein expression and signaling states, enabling quantitative links among microbial diversity, phenotypic breadth, and functional quality of vaccine-elicited immune cells [246,247].

Culturomics and Reductionist Models: While sequencing defines community structure and potential, functional validation needs experimental systems that directly test microbial activities. Culturomics, which is based on high-throughput anaerobic isolation and the use of matrix-assisted laser desorption ionization time-of-flight (MALDI-TOF) or genomic identification, has greatly increased the number of commensal strains available for mechanistic studies, allowing for systematic screening of bacteria that enhance dendritic-cell cytokine production, promote IgA class switching, or produce immunoregulatory metabolites [248]. Intestinal organoids and microfluidic “gut-on-a-chip” devices reproduce the epithelial architecture and can be co-cultured with immune cells and defined microbes, thus enabling a controlled examination of how microbial products or vaccine formulations affect barrier permeability, M-cell-mediated antigen transport, epithelial interferon responses, and the local cytokine gradients, and thereby linking in vitro precision with in vivo relevance [249,250].

Germ-Free, Gnotobiotic, and Humanized Models: Animal models remain indispensable for establishing causality in microbiota–vaccine interactions. Germ-free mice demonstrate that the absence of commensals leads to defective dendritic cell maturation, impaired germinal center formation, and reduced antibody titers, underscoring the requirement for microbial conditioning of immune competence [251,252,253]. Gnotobiotic mice colonized with defined consortia or single strains allow attribution of function to specific taxa, such as polysaccharide A-producing *Bacteroides fragilis* or SCFAs-generating *Clostridia*, in shaping T-cell polarization and humoral memory [254,255]. Humanized mice, reconstituted with human immune systems and FMT, further enable modeling of donor-specific vaccine responsiveness, providing a translational bridge to examine how “responder” versus “non-responder” microbial configurations influence outcomes after influenza, oral, or mRNA vaccination [256].

Systems Immunology and Computational Modeling. The multilayered complexity of host–microbe–vaccine interactions necessitates integrative analytical frameworks. Machine-learning approaches applied to multi-omics datasets identify minimal microbial, metabolic, and immune signatures predictive of seroconversion and memory durability, with supervised algorithms distinguishing responders from “non-responders” and unsupervised clustering uncovering ecological and immunological states associated with optimal protection [257,258]. Network analyses visualize crosstalk among microbial pathways, metabolite modules, and immune-cell circuits, highlighting central hubs such as SCFAs metabolism or T follicular helper cell programs. Causal-inference methods, including mediation analysis and Bayesian networks, move beyond association to infer mechanistic links [259]. More recently, agent-based models and digital-twin simulations of mucosal immunity incorporate diet, antibiotic exposure, and microbiota dynamics to forecast how interventions affect antibody longevity and memory T-cell pools, providing a platform for in silico optimization of personalized vaccination strategies and microbiome-targeted adjuvants [260].

Data Sharing and Harmonization: Robust translation of these approaches depends on standardized experimental design and open, interoperable data resources. International initiatives such as the Human Vaccines Project and global microbiome standardization consortia provide harmonized protocols for sampling, sequencing, immune phenotyping, and metadata reporting, enabling cross-study comparability [261,262]. Federated analysis frameworks allow multi-center integration of sensitive clinical and omics data without compromising privacy, facilitating large-scale meta-analyses across age groups, geographic regions, and vaccine platforms to identify reproducible microbial and immunological correlates of protection [263].

Collectively, these technological advances form a comprehensive toolkit for microbiota-aware vaccinology. Integrated metagenomic, metabolomic, and immune-phenotyping pipelines can identify microbial pathways that amplify germinal center reactions, sustain plasma cell survival, or promote balanced T-cell memory. Spatial and single-cell maps reveal where and how these signals act within tissues, while a computational model prioritizes the most effective dietary, probiotic, or adjuvant interventions for experimental validation (Table 5). Together, these approaches lay the foundation for precision vaccination strategies in which timing, formulation, and immune modulation are informed by an individual’s microbial ecology, thereby optimizing immunogenicity, durability, and protection equity across the lifespan.

## 10. Challenges, Knowledge Gaps, and Future Directions

Despite advances in elucidating the relationship between the microbiota and vaccine responses, significant challenges and knowledge gaps remain before these insights can be translated into practice. The primary issue is distinguishing causation from correlation; most human studies are observational, making it difficult to determine whether specific microbes or metabolites directly influence immunity or merely reflect confounding variables. This underscores the necessity for longitudinal, intervention-based studies employing controlled models to establish causality. Another challenge is the absence of standardized protocols for microbiome data collection and analysis, which hampers cross-study comparability and synthesis. As microbiome-based interventions approach clinical application, ethical and regulatory considerations become increasingly salient, particularly regarding the safety, stability, and oversight of live microbial and engineered therapies. The prospect of leveraging individual microbiome profiles to inform vaccine timing and formulation is promising, but it requires robust evidence, validated diagnostics, and equitable access. Addressing these challenges is essential for transitioning from descriptive associations to a precision vaccinology paradigm characterized by causal clarity, methodological standardization, regulatory preparedness, and personalized microbiome-informed vaccine strategies.

## 11. Conclusions

Accumulating evidence indicates that the human microbiota significantly contributes to interindividual variability in vaccine responses across the lifespan. The gut microbiome modulates both innate and adaptive immunity, maintains mucosal barrier integrity, and influences immune memory formation. Microbial development in early life affects pediatric vaccine outcomes, while diet and environmental exposures shape immune responses in adulthood, and age-related microbiota alterations may diminish vaccine efficacy in older adults. Advances in single-cell analysis and multi-omics technologies are elucidating the precise interactions between microbial products and the immune system at tissue and cellular levels. Although animal models and preliminary clinical trials suggest that microbiota modulation can influence vaccine effectiveness, further research is required to validate these findings in real-world human populations.

## Figures and Tables

**Table 1:** Factors modulating gut–vaccine interactions: microbial effects and evidence.

Modulator	Effect on Gut Microbiota	Immunological Consequence	Evidence for Impact on Vaccines	Refs.
Nutrition (dietary fiber, resistant starch, polyphenols, vitamins A/D, zinc, selenium)	↑ SCFAs-producing taxa (*Bifidobacterium*, *Roseburia*, *Faecalibacterium*); ↑ metabolic diversity; improved epithelial integrity	Enhanced dendritic-cell maturation, IgA class switching, and long-lived plasma cells	Nutritional status is associated with vaccine responsiveness; evidence that diet-induced microbiome changes directly mediate these effects remains limited	[80,81]
Antibiotics (broad-spectrum)	↓ diversity; depletion of SCFA producers; ↑ opportunists	Reduced PRR signaling, weaker plasmablast and germinal center responses	Adults on antibiotics show diminished influenza/mRNA vaccine titers; animal models confirm blunted responses	[82,83]
Other drugs (PPIs, NSAIDs, metformin, psychotropics)	Alter pH, bile acids, and metabolic networks	Context-dependent effects on barrier, APCs, and T/B-cell differentiation	Observational studies suggest altered serological outcomes; limited mechanistic work	[84,85]
Pathogen exposure and co-infections (enteric infections, helminths, HIV)	Barrier damage, ↑ inflammation, and altered community composition	Environmental enteropathy, Th2/Treg bias, impaired Th1/Tfh responses	Oral vaccines (rotavirus, cholera, OPV) are less effective in children with enteropathy or helminths; HIV blunts BCG and influenza responses	[86]
Geography and socioeconomic factors	Divergent “enterotypes” (*Bacteroides*- vs. *Prevotella*-dominated); sanitation and nutrition shape diversity	Geographic variation in mucosal immunity; chronic inflammation in low-resource settings	Lower efficacy of oral vaccines in LMICs vs. HICs; improved by WASH and nutrition programs	[87,88]
Probiotics (*L. rhamnosus* GG, *B. breve*, *S. boulardii*)	↑ beneficial taxa, SCFAs, and IgA-secreting plasma blasts	Adjuvant-like effects on mucosal and systemic responses	Trials show improved IgA or seroconversion for rotavirus, cholera, and influenza	[89,90,91]
Prebiotics (inulin, GOS, resistant starch)	Stimulate saccharolytic commensals; ↑ SCFAs	Enhanced barrier and germinal center support	Pilot studies suggest better oral vaccine titers; data are heterogeneous	[92,93]
Synbiotics (probiotic + prebiotic)	Synergistic colonization and metabolic output	Increased mucosal IgA, systemic titers	RCTs in infants show modest gains for rotavirus, polio	[59,94]
Postbiotics (SCFAs, indole derivatives, β-glucans, heat-killed bacteria)	Deliver immunomodulatory metabolites without live organisms	Boost dendritic-cell activity, T-cell polarization, and B-cell differentiation	Early trials and animal data indicate potential as safe adjuvants	[95,96]

**Abbreviations:** LMICs, Low- and middle-income countries; HICs, High-income countries; PRRs, Pattern recognition receptors; APCs, Antigen-presenting cells; Tfh, T-follicular helper cell; SCFAs, Short-chain fatty acids; RCT, Randomized controlled trial; Wash, Water-sanitation-hygiene interventions; Arrow ↑, Increases; Arrow ↓, Decreases; PPIs, Protein pump inhibitors; NSAIDs, Nonsteroidal anti-inflammatory Drugs; HIV, Human immunodeficiency viruses; *L., Lactobacillus*; GOS, Galactooligosaccharides; IgA, Immunoglobulin A; Th, T-helper cells; OPV, Oral polio vaccine; BCG, Bacille Calmette–Guerin.

**Table 2:** Evidence and interventions linking gut microbiota to vaccine responses.

Evidence/Intervention (Level of Evidence)	Model/Population	Microbial Effect	Immunological Outcome	Key Findings	Refs.
Infant/childbirth control (Human observational)	Birth cohorts (Bangladesh, Malawi, India, USA)	↑ *Bifidobacterium* spp., ↓ *Enterobacteriaceae*	Higher IgA titers, improved seroconversion (BCG, rotavirus, OPV)	Microbiota maturity and *Bifidobacterium* dominance predict oral vaccine success	[111,128,129]
Adult vaccine cohorts (Human observational)	Healthy adults (influenza, COVID-19 vaccines)	↑ SCFAs producers (*Faecalibacterium*, *Roseburia*)	Stronger plasma blast expansion, higher neutralizing antibody titers	Baseline diversity and SCFAs capacity correlate with vaccine durability	[130,131]
Older-adult cohorts (Human observational)	Long-term care residents (influenza, pneumococcal vaccines)	Retention of butyrate producers, ↓ dysbiosis	Better antibody persistence, improved T-cell recall	Frailty + dysbiosis linked to reduced protection	[132,133]
Probiotics/synbiotic RCTs in infants (Human randomized/Interventional)	Probiotic or synbiotic supplementation (South Asia, Africa)	↑ *Lactobacillus*, *Bifidobacterium*	↑ IgA, improved seroconversion (rotavirus, cholera, OPV)	Modest gains; strain- and dose-specific	[59,122,134]
Probiotics RCTs in infants (Human randomized/interventional)	Probiotic drinks or capsules (influenza vaccination)	Transient enrichment of lactic acid bacteria	↑ Antibody titers, improved mucosal IgA	Effect sizes variable; timing critical	[135,136]
Germ-free/antibiotic-treated mice (Mechanistic)	Mice, germ-free or post-antibiotic	Loss of microbial signals and metabolites	Impaired GC formation, reduced antibody titers	Recolonization or metabolite supplementation rescues responses	[137,138,139]
Gnotobiotic/humanized models (Mechanistic)	Mice colonized with defined or human microbiota	Defined consortia with SCFA producers	Improved IgA and memory T-cell differentiation	Captures donor-specific vaccine outcomes	[140,141]
Probiotics	Infants, children, adults	Enrich *Lactobacillus*, *Bifidobacterium*	↑ Mucosal IgA, enhanced DC maturation	Shown to improve oral and parenteral vaccine responses	[135,136]
Synbiotics	Infants, low-resource settings	Probiotic colonization + prebiotic fuel	↑ SCFAs output, ↑ IgA titers	Modest improvement in oral vaccine efficacy	[142,143,144,145]
Postbiotics without established vaccine RCT evidence	Preclinical models; early-phase human trials	Direct delivery of SCFAs, indoles, β-glucans	Improved DC antigen presentation, B/T-cell differentiation	Safe, stable, promising as standardized adjuvants	[22,23,146]
FMT for improving vaccine responses	Immunocompromised or dysbiotic hosts	Restores diversity, SCFA production	Baseline immune tone recovery	Early studies are promising; vaccine outcomes are not yet well tested	[147]
Engineered microbes	Preclinical (mice, pigs)	Attenuated strains delivering antigen + adjuvant	Local antigen secretion, PRR activation	Enhanced protection in respiratory/enteric vaccine models	[148]

**Abbreviations:** PRRs, Pattern recognition receptors; SCFAs, Short-chain fatty acids; FMT, Fecal microbiome transplant; Arrow ↑, Increases; Arrow ↓, Decreases; PPIs, Protein pump inhibitors; NSAIDs, Nonsteroidal anti-inflammatory Drugs; DC, Dendritic cells; *L., Lactobacillus*; GOS, Galactooligosaccharides; IgA, Immunoglobulin A; Th, T-helper cells; OPV, Oral polio vaccine; BCG, Bacille Calmette–Guerin; COVID-19, COronaVIrus Disease of 2019; GC, Germinal Center.

**Table 3:** Comparative influence of the gut microbiota on different vaccine platforms.

Vaccine Platform	Route/Examples	Strength of Microbiota–Vaccine Evidence	Key Microbial Mechanisms	Evidence/Key Findings	Refs.
Live-attenuated vaccines (LAVs)	Oral: rotavirus, OPV, cholera, typhoid. Intranasal: LAIV (influenza), RSV	High	Replication at mucosal surfaces requires intact commensals and barrier health; SCFA producers and *Bifidobacterium* enhance antigen uptake and IgA; dysbiosis and enteropathy reduce “take.”	Infants with abundant *Bifidobacterium* show better IgA titers to rotavirus and OPV; nasal commensals (*Corynebacterium*, *Dolosigranulum*) correlate with LAIV responses.	[12,163,164,169,170]
Inactivated vaccines	Parenteral influenza, polio (IPV), hepatitis A	Moderate	Baseline immune tone set by SCFA producers; microbial metabolites support dendritic-cell metabolism, plasma blast expansion, and long-lived plasma cells	Adults on antibiotics show blunted plasma blast response to influenza; diversity predicts antibody persistence.	[21,171]
Subunit and conjugate vaccines	Tdap; Hib and pneumococcal conjugates	Moderate	Microbiota enhance Tfh/B-cell help for polysaccharides; metabolic cues (butyrate, indoles) improve memory.	Associations between butyrate producers and sustained antibody titers to conjugates	[172]
mRNA vaccines	COVID-19 (BNT162b2, mRNA-1273), experimental mRNA influenza/Rabies	Context-dependent	Commensals calibrate innate sensors (TLR7/8, RIG-I), promote balanced interferon tone, and sustain germinal center B cells.	Antibiotic use linked to lower neutralizing Ab and shorter plasmablast persistence in COVID-19 vaccinees.	[9,18,173,174]
Viral-vector vaccines	Adenoviral or MVA COVID-19, Ebola	Context-dependent	Microbiota modulate early cytokines and tissue-resident APC pools; SCFAs producers support CD8^+^ T-cell expansion.	Greater diversity predicts stronger spike-specific responses to adenoviral COVID-19 vaccines.	[171,175]
Adjuvants and delivery systems	Alum, MF59, CpG, lipid nanoparticles, microneedles	Variable (all platforms)	Microbial products prime PRRs and APC metabolism; SCFAs/indoles modulate chemokines and T/B-cell differentiation	PRR agonist adjuvants interact with baseline commensal signals; alum/MF59 efficacy shaped by immune tone	[176,177]

**Abbreviation:** LAVs, Live-attenuated vaccines; OPV, Oral polio vaccine; LAIV, Live-attenuated influenza vaccine; RSV, Respiratory syncytial virus; IPV, Inactivated polio vaccine; Hib, Haemophilus influenzae type b; mRNA, Messenger ribonucleic acid; MVA, Modified vaccinia Ankara; SCFA(s), Short-chain fatty acid(s); IgA, Immunoglobulin A; Ab, Antibody; APC(s), Antigen-presenting cell(s); PRR(s), Pattern-recognition receptor(s); TLR, Toll-like receptor; RIG-I, Retinoic acid-inducible gene I; Tfh cell(s), T follicular helper cell(s); CD8^+^ T cell(s), Cluster of differentiation 8-positive T lymphocyte(s).

**Table 4:** Microbiota–vaccine interaction in special populations.

Population	Typical Microbiota Features	Vaccine-Related Challenges	Evidence and Key Findings	Potential Strategies	Refs.
Preterm and low-birthweight neonates	Immature, low-diversity microbiota; ↑ *Enterobacteriaceae*, *Staphylococcus*; ↓ *Bifidobacterium*, SCFAs	Weak innate signaling; impaired germinal centers; low seroconversion and short antibody persistence (e.g., HBV, BCG, pertussis)	Cohorts show reduced titers vs. term infants; microbial maturity correlates with oral vaccine IgA.	Promote maternal milk feeding; limit antibiotics; targeted probiotics/synbiotics; adjusted dosing schedules.	[179,180,181]
Pregnant and lactating women	Gestational shifts: ↑ Proteobacteria and Actinobacteria; ↑ metabolic turnover	Variability in vaccine titers (Tdap, influenza, COVID-19); microbiota influence transfer of antibodies and SCFAs via placenta/breast milk	Preliminary studies link fiber-rich diets/probiotics with higher vaccine IgG and IgA in milk.	Nutritional optimization; probiotic supplementation (strain-specific); minimize peripartum antibiotics	[182,183,184,185]
Transplant recipients	Loss of obligate anaerobes; ↑ *Enterococcus*, *Candida*; antibiotic pressure	Reduced antigen presentation and seroconversion (influenza, HBV, COVID-19); safety concerns with live vaccines	Dysbiosis severity predicts poor titers post-transplant	Diet rich in fiber; SCFAs/postbiotic supplementation; carefully selected probiotics (after safety assessment); early vaccination pre-immunosuppression	[186,187,188,189]
HIV Patients	Dysbiosis with ↓ butyrate-producers, ↑ Proteobacteria; barrier disruption	Suboptimal titers (HPV, HBV, pneumococcal); persistent inflammation blunts germinal centers	ART improves but does not normalize responses; gut leakiness correlates with poor vaccines	Gut barrier repair, prebiotics, SCFAs-rich diets; adjuvanted vaccines; early immunization	[190,191]
Patients on biologics (e.g., anti-TNF, IL-17)	Cytokine-targeted therapy shifts gut flora (↓ Firmicutes, ↑ Proteobacteria)	Blunted response to influenza, COVID-19 boosters	Observational data show lower Ab titers vs. untreated controls	Vaccinate before therapy or during quiescence; consider booster/adj. formulations; monitor microbiota	[100,174,192,193,194]
Obesity and T2D	Reduced diversity; ↑ LPS-producing Enterobacteriaceae; ↓ SCFAs output	“Metaflammation” impairs dendritic-cell metabolism and B/T-cell memory; faster Ab decay	Lower seroconversion and waning titers in obese adults	Weight control; fiber/polyphenols; SCFAs supplementation; probiotics; prebiotics	[7,42,79,195,196,197,198,199,200,201,202,203,204,205]
IBD (Crohn’s, UC)	Loss of *Faecalibacterium prausnitzii* and Clostridia XIVa; ↑ Proteobacteria; mucosal inflammation	Avoid live vaccines during flares; titers may be blunted on steroids or biologics.	Stable patients mount adequate responses; dysbiosis may affect durability	Vaccinate in remission; manage inflammation; microbiota support (diet, synbiotics)	[206,207]

**Abbreviation:** Ab, antibody; ACTH, adrenocorticotropic hormone; ART, antiretroviral therapy; BCG, Bacillus Calmette-Guérin; B cells, B lymphocytes; Arrow ↑, Increases; Arrow ↓, Decreases; COVID-19, coronavirus disease 2019; HBV, hepatitis B virus; HIV, human immunodeficiency virus; IBD, inflammatory bowel disease; IgA, immunoglobulin A; IgG, immunoglobulin G; IL, interleukin; LPS, lipopolysaccharide; SCFAs, short-chain fatty acids; T2D, type 2 diabetes; T cells, T lymphocytes; Tdap, tetanus–diphtheria–acellular pertussis vaccine; TNF, tumor necrosis factor; UC, ulcerative colitis.

**Table 5:** Technological and methodological tools for studying gut–vaccine interaction.

Technology/Method	Core Principle	Key Insights for Gut–Vaccine Research	Advantages	Limitations/Challenges	Refs.
Shotgun metagenomics	Sequencing all the DNA in a sample to identify taxa and functional genes	Links specific species and pathways (e.g., butyrate synthesis, tryptophan metabolism) to antibody titers and seroconversion	High taxonomic and functional resolution; detects bacteria, archaea, viruses, fungi	Costly for large cohorts; bioinformatic complexity; may miss low-abundance transcriptionally active species	[202,264,265]
16S rRNA sequencing	Amplifies conserved rRNA regions for bacterial profiling	Broad associations between microbiota diversity and vaccine efficacy	Inexpensive; scalable to large studies	Limited resolution (genus level); no functional data	[198,203,266,267,268,269,270]
Metatranscriptomics and Metaproteomics	Quantify microbial gene expression and protein abundance	Captures real-time metabolic activity and adjuvant-like molecules during vaccination	Functional insight; time-resolved	RNA/protein instability; need deep sequencing; difficult in low biomass samples	[232,233,234]
Metabolomics (untargeted/targeted)	Mass spectrometry or NMR to measure metabolites	Identifies SCFAs, bile acids, and indoles correlating with immune priming and memory	Bridges diet, microbes and immunity; quantitative	Requires careful sample handling; limited structural annotation	[236,237]
Spatial transcriptomics and tissue imaging	Localize host gene expression and cell markers in tissues	Maps where microbial signals and antigens shape DC/T-cell responses in GALT and Peyer’s patches	Maintains tissue architecture; high multiplexing	Expensive, complex analysis pipelines	[238,239,240]
Single-cell RNA-seq/ATAC-seq	Profiles the transcriptomes or chromatin of individual immune cells	Defines states of DCs, Tfh, and B cells in relation to microbiota composition	Resolves heterogeneity; links clonal expansion to baseline flora	High cost; need computational expertise	[241,242,243,244,245]
High-dimensional cytometry (CyTOF, spectral flow)	Measures >30 markers on single cells	Associates microbial diversity with the breadth of vaccine-elicited cell phenotypes	Multiplexed immune profiling	Requires expert panel design and data interpretation	[246,247]
Culturomics	High-throughput anaerobic culture + MALDI-TOF ID	Isolates candidate commensals that enhance vaccine responses	Yields live strains for mechanistic testing	Labor-intensive; not all taxa are culturable	[248]
Organoids and gut-on-chip models	Three-dimensional epithelial culture ± immune/microbial co-culture	Test how microbes/metabolites influence barrier, M-cell sampling, antigen transport	Controlled environment; reductionist	Limited immune complexity; may lack systemic readouts	[249,250]
Germ-free/gnotobiotic animals	Mice reared sterile or colonized with defined microbiota	Show causality: loss or gain of microbes/metabolites alters antibody and T-cell memory	Robust mechanistic platform	Species differences; ethical and cost issues	[251,252,253]
Humanized mouse models	Mice engrafted with the human immune system and microbiota	Recreate donor-specific vaccine responses in vivo	Translational bridge	Engraftment variability; high expense	[256]
Machine learning and network modeling	Algorithms on multi-omics datasets	Predict responders, prioritize adjuvant targets, and simulate interventions.	Integrates complex data; hypothesis generation	Risk of overfitting; needs large, well-annotated cohorts	[252,253,254]
Digital twins/agent-based simulations	Computational replicas of host–microbe–immune systems	Forecast effects of diet, antibiotics, or microbes on vaccine efficacy	Predictive; guides trial design	Requires curated datasets; model validation is essential	[260]

**Abbreviation:** 16S rRNA, 16S Ribosomal Ribonucleic Acid; ATAC-seq, Assay for Transposase-Accessible Chromatin using sequencing; CyTOF, Cytometry by Time-of-Flight; DCs, Dendritic Cells; GALT, Gut-Associated Lymphoid Tissue; MALDI-TOF, Matrix-Assisted Laser Desorption/Ionization-Time of Flight; NMR, Nuclear Magnetic Resonance; RNA-seq, Ribonucleic Acid Sequencing; SCFAs, Short-Chain Fatty Acids; Tfh cells, T Follicular Helper Cells; APCs, Antigen-Presenting Cells; IgA, Immunoglobulin A; PRRs, Pattern-Recognition Receptors; LPS, Lipopolysaccharide.

## Data Availability

No new data were created or analyzed in this study. Data sharing is not applicable to this article.
